# The triglyceride-glucose–waist-to-height ratio is the strongest predictor of reduced kidney function in elderly hypertensive patients

**DOI:** 10.3389/fendo.2026.1789763

**Published:** 2026-06-15

**Authors:** Jiaying Ling, Shu Xie, Yanyan Zhang, Danxiang Chen, Changwei Lü, Gongcheng Wang, Xueqin Li

**Affiliations:** 1 Department of General Practice, The Affiliated Huaian No.1 People’s Hospital of Nanjing Medical University, Huaian, Jiangsu, China; 2 Department of Urology, The Affiliated Huaian No.1 People’s Hospital of Nanjing Medical University, Huaian, Jiangsu, China

**Keywords:** elderly, estimated glomerular filtration rate, hypertension, insulin resistance, triglyceride-glucose-waist-to-height ratio

## Abstract

**Background:**

The triglyceride-glucose index (TyG) and its derivatives, such as the TyG-waist-to-height ratio (TyG-WHtR), provide reliable surrogate measures for estimating insulin resistance (IR). Their associations with renal function in hypertensive patients remain unclear. This study examined the associations of TyG-WHtR and its related indices with estimated glomerular filtration rate (eGFR) in a community-based elderly hypertensive population.

**Methods:**

This cross-sectional study assessed the clinical indicators, TyG-related indices, and eGFR in a cohort of 3,764 hypertensive adults aged ≥65 years from Huaian, China, employing restricted cubic spline models (RCS), logistic regression, spearman correlation, and receiver operating characteristic (ROC) curve analyses to evaluate the associations and predictive utility of TyG-WHtR and related parameters for identifying impaired renal function, defined by reduced eGFR.

**Results:**

A total of 612 participants (16.25%) had reduced eGFR. Significant predictors of reduced eGFR included elevated TyG-WHtR and related indices, with TyG-WHtR showing a particularly robust association in both pre- and post-adjustment analyses. These indices correlated negatively with high-density lipoprotein cholesterol (HDL-C), and positively with measures of adiposity, fasting plasma glucose (FPG), and non-HDL cholesterol. Significant interactions were found for gender, age, and alcohol consumption. TyG-WHtR alone had an AUC of 0.596, but after adjustment its predictive ability improved to 0.864, demonstrating superior performance compared to TyG, TyG-body mass index (TyG-BMI), and TyG-waist circumference (TyG-WC).

**Conclusion:**

Elevated TyG-WHtR is an independent risk factor for the decline in eGFR in elderly patients with hypertension, demonstrating superior predictive value compared to other TyG-related indices. TyG-WHtR may serve as a useful marker for early identification of renal dysfunction in this population.

## 1 Introduction

With the rapid progression of population aging, chronic diseases, functional decline, and multimorbidity among older adults have become significant challenges for China’s public health system, posing serious threats to the overall health of the people. Data from the Seventh National Population Census (2020) indicate that the population aged 65 and above in China stood at 190 million, accounting for 13.5% of the total population (1). In China, the prevalence of hypertension increases significantly with age and is particularly high among adults aged ≥65 years (2). From 2002 to 2019, hypertension rates among Chinese adults 60 and older rose from 48.7% to 64.9%, an average annual increase of 2.3% (3). Hypertension is a significant risk factor that markedly elevates the likelihood of developing chronic kidney disease (CKD) and is closely associated with both its onset and progression. (4, 5). Approximately 18.2% of Chinese adults with hypertension also have CKD, and sustained hypertension can reduce the estimated glomerular filtration rate (eGFR), thereby accelerating CKD development (6, 7). Therefore, the timely detection of populations at risk for hypertensive nephropathy is essential to inform strategies for preventing hypertension-related renal injury, representing a key clinical objective.

A condition defined by a decrease in cellular insulin sensitivity and responsiveness, insulin resistance (IR) elevates hypertension risk and fosters renal damage. This occurs via mechanisms that include disruption of the glomerular filtration barrier, heightened endothelial permeability, inflammation, and oxidative stress (8, 9). The triglyceride-glucose index (TyG), a novel indicator for evaluating IR, has been demonstrated to outperform the homeostatic model assessment of insulin resistance(HOMA-IR) in assessing IR (10). Moreover, a series of novel indices that integrate TyG with basic anthropometric parameters—namely, the triglyceride-glucose-waist circumference (TyG-WC), triglyceride-glucose-body mass index (TyG-BMI), and triglyceride-glucose-waist-to-height ratio (TyG-WHtR)—have demonstrated considerable clinical value. In community residents in southern China, TyG-WHtR has been shown to provide better diagnostic value for CKD than other TyG-related indices; Nonetheless, the TyG index maintained a superior association with CKD when compared to all other indices examined (11). In patients with type 2 diabetes, the TyG index and the triglyceride-to-high-density lipoprotein cholesterol ratio (TG/HDL-C ratio) demonstrated the strongest correlation with diabetic nephropathy (12). In the Chinese population aged 45 years and above with normal renal function, TyG-WC has been identified as the most effective IR index for predicting a rapid decline in renal function (13). However, a systematic comparison of the associations and predictive capacities between TyG-WHtR and other related parameters (namely TyG-WC, TyG, and TyG-BMI) for forecasting the decline in eGFR among an elderly hypertensive community population is still lacking. Consequently, this research aimed to examine the relationships between the TyG-WHtR along with its associated parameters and eGFR among community-based hypertensive aged 65 or older in Huaian City, Jiangsu Province. The goal was to provide insights for enhancing primary care approaches for hypertensive nephropathy.

## 2 Materials and Methods

### 2.1 Research subjects

This cross-sectional study employed a stratified sampling method. A total of 20 community health service centers were selected as study units from four districts—Qingjiangpu District, Huaiyin District, Huaian District, and Hongze District—in Huaian City, Jiangsu Province. Elderly hypertensive patients under chronic disease management who underwent health examinations at the study units between January and December 2024 were included. Inclusion criteria: (1). hypertension diagnosis met the “Guidelines for Hypertension Management in Primary Care (2019) (14); (2). age ≥ 65 years. Exclusion criteria: (1). patients with tumor, decompensated cardiopulmonary function, severe diseases, or other severe complications; (2). those with primary kidney disease or undergoing hemodialysis, peritoneal dialysis, or kidney transplantation; (3). those with a history of mental illness; (4). those presenting significant communication barriers or deemed uncooperative after evaluation; (5). those residing outside the catchment area of the selected community health service center; (6). those with incomplete clinical data; (7). patients were excluded if their aspartate aminotransferase (AST) or alanine aminotransferase (ALT) levels exceeded 120 U/L, or if their total bilirubin (TBil) level exceeded 34.2 μmol/L.

### 2.2 Research methods

#### 2.2.1 Clinical data collection

1. Participants’ socio-demographic profiles, including key personal characteristics (e.g., name, age, and gender) and lifestyle habits (namely physical activity, smoking, and alcohol consumption), were obtained via a structured questionnaire.
2. Medical history: Data on comorbidities, hypertension grade, and cardiovascular risk stratification were obtained through standardized questionnaires and review of prior medical records.
3. Trained medical staff performed physical examinations to measure participants’ height, body weight, and waist circumference (WC), from which body mass index (BMI [=weight (kg)/height (m)²]) and waist-to-height ratio (WHtR [=WC (cm)/height (cm)]) were calculated, while systolic and diastolic blood pressure (SBP, DBP) along with resting heart rate (RHR) were also assessed.
4. Following a minimum 8-hour overnight fast, venous blood was drawn for a comprehensive panel of laboratory analyses, including platelet count (Plt), hemoglobin (Hb), white blood cell count (WBC), ALT, low-density lipoprotein cholesterol (LDL-C), fasting plasma glucose (FPG), AST, total cholesterol (TC), serum creatinine (SCr), TBil, triglycerides (TG), high-density lipoprotein cholesterol (HDL-C), and urinary protein.

#### 2.2.2 Blood pressure measurement

Blood pressure was measured by trained and experienced medical personnel using an upper-arm electronic sphygmomanometer (model HBP-1300, Omron (Dalian) Co., Ltd.), which complies with the standards of the Association for the Advancement of Medical Instrumentation (AAMI). Before measurement, participants were asked whether they had consumed coffee, alcohol, or engaged in vigorous physical activity within the preceding 30 min, and it was confirmed that they were emotionally calm. They were then instructed to sit quietly and rest for 5–10 min and to empty their bladder. Three consecutive seated upper arm blood pressure readings were obtained using a cuff size of 22–32 cm, with the participant’s arm maintained at heart level. Each measurement was taken at 1–2 minintervals. The average of the final two measurements was recorded as the blood pressure value. If the last two systolic or diastolic readings obtained differed by more than 5 mmHg, an additional measurement was taken, and the mean of the final three values was calculated and recorded.

#### 2.2.3 Calculation of TyG-WHtR, related indices, and eGFR

(1) $$\text{TyG}=\ln\left(\frac{\text{TG}\left(\text{mg}/\text{dL}\right)\times \text{FPG}\left(\text{mg}/\text{dL}\right)}{2}\right)$$

(2) $$\text{TyG}-\text{BMI}=\text{TyG}\times \left(\frac{\text{weight}\left(\text{kg}\right)}{\text{height}{\left(\text{m}\right)}^{2}}\right)$$

(3) TyG−WC=TyG×WC(cm)

(4) $$\text{TyG}-\text{WHtR}=\text{TyG}\times \left(\frac{\text{WC}\left(\text{cm}\right)}{\text{height}\left(\text{cm}\right)}\right)$$

eGFR was estimated using the FAS Scr formula (15). For females,

(5) $$\text{eGFR}\left(\text{mL}\cdot {\text{min}}^{-1}\cdot {\left(1.73\;{\text{m}}^{2}\right)}^{-1}\right)=\frac{107.3}{\left(\text{Scr}\left(\text{mg}/\text{dL}\right)/0.7\right)}\times 0.988^{\left(\text{age}-40\right)}$$

for males,


$$\text{eGFR}\left(\text{mL}\cdot \min^{-1}\cdot {\left(1.73{\text{ m}}^{2}\right)}^{-1}\right)=\frac{107.3}{\left(\text{Scr}\left(\text{mg}/\text{dL}\right)/0.9\right)}\times 0.988^{\left(\text{age}-40\right)}$$

#### 2.2.4 Definitions

1. Hypertension was diagnosed when three or more independent office measurements yielded a blood pressure of at least 140/90 mmHg (1 mmHg = 0.133 kPa) in the absence of antihypertensive drug therapy. Individuals with a documented history of hypertension who were currently receiving antihypertensive drug treatment were also classified as having hypertension.
2. Individuals were considered smokers if they had smoked at least one cigarette per day for a minimum of six months.
3. Alcohol consumption was defined as the habitual intake of more than 30 grams per day or 210 grams per week on average, sustained for a minimum period of six months.
4. Physical exercise referred to physical activity ≥3 times/week, with each session ≥30 min.
5. Hypertension grading and cardiovascular risk stratification followed the Guidelines for Hypertension Management in Primary Care (2019) (14). Hypertension was categorized into grades 1–3, and cardiovascular risk was classified as low, moderate, high, or very high based on blood pressure level, risk factors, target organ damage, clinical complications, and diabetes.
6. Comorbidities included diabetes, coronary heart disease, and stroke.
7. An eGFR below 60 mL/min/1.73 m² constituted decreased kidney function. Participants whose current eGFR had decreased by<25% compared with the most recent testing within one year were included.

### 2.3 Statistical methods

All statistical analyses were conducted using SPSS 22 and RStudio (R version 4.5.0). Samples with missing clinical data were excluded using listwise deletion. Continuous variables following a normal distribution were summarized as mean ± SD and compared using Student’s t-test, while those with a non-normal distribution were reported as median with IQR and compared via the Mann-Whitney U test. Categorical data were summarized as frequency (percentage). Unordered categorical variables were analyzed using the χ² test or Fisher’s exact test, the latter being employed when the conditions for the χ² test were not satisfied. For ordered categorical variables, the Cochran–Armitage trend test was applied, with Fisher’s exact test used where its assumptions were violated. The Spearman correlation test was employed for continuous variables that deviated from normal distribution. Associations among TyG-WHtR, related indicators, and metabolic parameters were assessed using Spearman correlation analysis. The relationship between eGFR and TyG-WHtR, as well as related indicators, was examined using a binary logistic regression model. Participants were further divided into four groups based on the median values of TyG and WHtR, and a multivariate logistic regression model was used to assess the association between each group and renal function decline. The synergistic effect of the two factors on kidney function was further examined by using multiplicative interaction terms and the relative excess risk due to interaction (RERI), with the fully adjusted model (Model 4) applied. After the linear relationship was verified through curve fitting, logistic regression was performed to quantify the risk of decreased eGFR associated with TyG-WHtR and its related indicators. The capability of TyG-WHtR and related indicators for predicting decreased eGFR was assessed through receiver operating characteristic (ROC) curve analysis, with statistical comparisons performed using DeLong’s test. A two-tailed approach was adopted for all statistical tests, with P< 0.05 set as the significance threshold.

## 3 Results

### 3.1 Clinical characteristics of the community-based elderly hypertensive population aged 65 years or older

A total of 3,765 participants were enrolled in this study, including 612 (16.25%) in the eGFR decline group and 3,153 (83.75%) in the eGFR normal group. Compared with the eGFR normal group, the eGFR decline group showed significantly lower smoking rates, alcohol consumption rates, physical exercise rates, DBP, height, weight, Hb, ALT, AST, TBil, and HDL-C levels (all *P* < 0.05). In contrast, the decline group was characterized by a significantly older age, higher values of WC, BMI, WBC, FPG, TG, SBP, TyG, TyG-BMI, TyG-WC, and TyG-WHtR, a greater proportion of individuals at very high hypertension risk, a greater proportion of females, more comorbidities, and a higher rate of positive proteinuria (all *P* < 0.05). No significant between-group differences were observed for RHR, Plt, TC, hypertension grade, or LDL-C (all *P* > 0.05) (**Table 1**). Specific calculation formulas are given in Equations 1–5.

**Table 1:** Clinical characteristics of the community-dwelling elderly hypertensive population aged 65 or older in the eGFR normal group and eGFR decline group.

Variable	eGFR normal group (3153)	eGFR decline group (612)	*P* value	χ2/Z/t
Age, year	71.0 (68.0, 75.0)	79.0 (75.0, 83.0)	<0.001	25.168
Height, cm	161.5 (155.0, 168.0)	157.6 (152.5, 163.5)	<0.001	-9.156
Weight, kg	67.3 (60.2, 74.5)	64.8 (58.7, 72.6)	<0.001	-4.538
WC, cm	89.0 (84.0, 95.0)	90.0 (85.0, 97.0)	0.004	2.895
BMI, kg/m2	25.8 (23.8, 27.9)	26.0 (24.0, 28.3)	0.031	2.158
RHR, bpm	72.0 (66.0, 80.0)	73.0 (65.0, 81.0)	0.139	1.477
Hb, g/L	140.0 (131.0, 149.0)	130.0 (120.0, 140.0)	<0.001	-14.951
WBC, 10^9/L	6.0 (5.1, 7.0)	6.4 (5.4, 7.4)	<0.001	5.522
Plt, 10^9/L	193.0 (163.0, 226.0)	188.0 (158.8, 227.0)	0.257	-1.134
FPG, mmol/L	6.2 (5.6, 7.5)	6.3 (5.7, 7.9)	0.001	3.285
ALT, u/L	20.7 (16.0, 28.1)	17.1 (12.6, 22.6)	<0.001	-10.221
AST, u/L	22.3 (19.0, 27.0)	21.1 (18.0, 25.7)	<0.001	-4.618
TBil,μmol/L	14.3 (11.6, 17.7)	13.6 (10.8, 16.6)	<0.001	-3.822
TC, mmol/L	4.7 (4.0, 5.4)	4.7 (4.0, 5.6)	0.443	-0.768
TG, mmol/L	1.4 (1.0, 2.0)	1.5 (1.1, 2.2)	<0.001	3.484
LDL-C, mmol/L	3.1 (2.5, 3.6)	3.1 (2.4, 3.9)	0.350	0.935
HDL-C, mmol/L	1.3 (1.1, 1.6)	1.3 (1.1, 1.5)	0.046	-1.994
SBP, mmHg	147.0 (135.0, 160.0)	150.0 (138.0, 162.0)	0.024	2.250
DBP, mmHg	86.0 (79.0, 92.0)	81.0 (74.0, 89.2)	<0.001	-8.142
TyG	8.9 (8.5, 9.3)	9.0 (8.6, 9.5)	<0.001	3.949
TyG-BMI	229.8 (208.2, 252.6)	235.5 (212.0, 263.6)	<0.001	3.821
TyG-WC	793.9 (732.5, 860.8)	819.4 (745.5, 887.6)	<0.001	4.503
TyG-WHtR	4.9 (4.5, 5.4)	5.2 (4.6, 5.7)	<0.001	7.560
Gender			<0.001	-8.455
Female	1472 (46.7)	400 (65.4)		
Male	1681 (53.3)	212 (34.6)		
Smoking			<0.001	-5.265
No	2720 (86.3)	575 (94.0)		
Yes	433 (13.7)	37 (6.0)		
Alcohol consumption			<0.001	-8.048
No	2293 (72.7)	539 (88.1)		
Yes	860 (27.3)	73 (11.9)		
Hypertension grading			0.0958	-1.665
Level 1	2240 (71.04)	427 (69.8)		
Level 2	710 (22.52)	126 (20.6)		
Level 3	203 (6.44)	59 (9.6)		
Hypertension risk level			<0.001	-6.648
Moderate risk	1396 (44.3)	199 (32.5)		
High risk	651 (20.6)	110 (18.0)		
Very high risk	1106 (35.1)	303 (49.5)		
Comorbidity			<0.001	6.808
No	2187 (69.4)	338 (55.2)		
Yes	966 (30.6)	274 (44.8)		
Urine protein			<0.001	4.794
No	2507 (79.5)	433 (70.8)		
Yes	646 (20.5)	179 (29.2)		
Physical exercise			0.003	-2.997
No	1543 (48.9)	340 (55.6)		
Yes	1610 (51.1)	272 (44.4)		

Values were represented by median (IQR) for continuous variables and frequency (percentage) for categorical variables. WC, waist circumference; BMI, body mass index; RHR, resting heart rate; Hb, hemoglobin; WBC, white blood cell; Plt, platelet; FPG, fasting plasma glucose; ALT, alanine aminotransferase; AST, aspartate aminotransferase; TBil, total bilirubin; TC, total cholesterol; TG, triglyceride; LDL-C, low density lipoprotein; HDL-C, high density lipoprotein cholesterol; SBP, systolic blood pressure; DBP, diastolic blood pressure; TyG, triglyceride-glucose index; TyG-BMI, triglyceride-glucose-body mass index; TyG-WC, triglyceride-glucose-waist circumference; TyG-WHtR, triglyceride-glucose-waist-to-height ratio; IQR, interquartile range.

### 3.2 The association between TyG-WHtR-related parameters and lowered eGFR

The presence of decreased eGFR was used as the dependent variable (coded as no = 0, yes = 1). Model 1 did not incorporate any adjustment. In Model 2, covariates for gender and age were included. Model 3 extended these adjustments by additionally accounting for smoking status, alcohol intake, comorbidities, hypertension risk category, and level of physical activity. For Model 4, further variables such as DBP, SBP, urine protein, HDL-C, TBil, ALT, AST, Hb, and WBC were added to the existing set of covariates. Assessments for multicollinearity in Models 3 and 4 indicated low collinearity, as all independent variables exhibited a variance inflation factor (VIF) below 5 (**Supplementary Tables 1**-**4**). The association between TyG-WHtR and its related indicators (continuous variables) with the decline in renal function among the community elderly hypertensive population was analyzed using a multivariate logistic regression model. In Model 1, which did not adjust for confounding factors, TyG-WHtR exhibited the strongest association with the risk of reduced eGFR (*OR* = 1.70, *95% CI*: 1.49–1.93). Its effect size exceeded those of TyG (*OR* = 1.34, *95% CI*: 1.17–1.53), TyG-WC (*OR* = 1.00, *95% CI*: 1.00–1.00) and TyG-BMI (*OR* = 1.00, *95% CI*: 1.00–1.01), with all comparisons showing statistical significance (all *P* < 0.001). Following multivariable adjustment (Model 4), higher TyG-WHtR levels were independently associated with higher odds of decreased eGFR (*OR* = 1.49, *95% CI*: 1.25-1.79, *P* < 0.001). Notably, the magnitude of risk linked to TyG-WHtR was greater compared to the associations observed for TyG-BMI (*OR* = 1.01, *95% CI*: 1.01-1.01, *P* < 0.001) and TyG-WC (*OR* = 1.00, *95% CI*: 1.00-1.00, *P* < 0.001) (**Table 2**).

**Table 2:** Multivariate logistic regression analysis of TyG-WHtR and its related indicators associated with decreased eGFR.

Variable	Model 1		Model 2		Model 3		Model 4	
	*OR* (*95%CI*)	*P* value	*OR* (*95%CI*)	*P* value	*OR* (*95%CI*)	*P* value	*OR* (*95%CI*)	*P* value
TyG	1.34 (1.17-1.53)	<0.001	1.41 (1.21-1.66)	<0.001	1.29 (1.09-1.52)	0.003	1.51 (1.25-1.82)	<0.001
TyG-BMI	1.00 (1.00-1.01)	<0.001	1.01 (1.00-1.01)	<0.001	1.01 (1.00-1.01)	<0.001	1.01 (1.01-1.02)	<0.001
TyG-WHtR	1.70 (1.49-1.93)	<0.001	1.39 (1.19-1.62)	<0.001	1.34 (1.14-1.57)	<0.001	1.49 (1.25-1.79)	<0.001
TyG-WC	1.00 (1.00-1.00)	<0.001	1.00 (1.00-1.00)	<0.001	1.00 (1.00-1.00)	<0.001	1.00 (1.00-1.01)	<0.001

TyG, triglyceride-glucose index; TyG-BMI, triglyceride-glucose-body mass index; TyG-WC, triglyceride-glucose-waist circumference; TyG-WHtR, triglyceride-glucose-waist-to-height ratio.

TyG-WHtR and its related indicators were categorized according to quartile levels and divided from low to high into Q1 (TyG-WHtR< 4.55, TyG< 8.50, TyG-BMI< 208.46, TyG-WC< 734.14), Q2 (TyG-WHtR 4.55–<4.94, TyG 8.50–<8.89, TyG-BMI 208.46–<230.9, TyG-WC 734.14–<797.12), Q3 (TyG-WHtR 4.94–<5.42, TyG 8.89–<9.33, TyG-BMI 230.9–<254.38, TyG-WC 797.12–<865.50), and Q4 group (TyG-WHtR ≥ 5.42, TyG ≥ 9.33, TyG-BMI ≥ 254.38, TyG-WC ≥ 865.50), with Q1 as the baseline. A significantly increased risk of decreased eGFR was observed in the Q3 group of Model 1 (*OR* = 1.44, *95%CI*: 1.11–1.86, *P* = 0.006), indicating that TyG-WHtR had an earlier warning value compared with other indices. In contrast, no consistent statistical significance was found for TyG, TyG-WC, or TyG-BMI in the Q3 group (Model 1: all *P* values> 0.05). In Model 1, the most significant risk increase was observed in the Q4 group of TyG-WHtR, showing a 118% higher risk compared with the Q1 group (*OR* = 2.18, *95%CI*: 1.71–2.80, *P* < 0.001). This increase was greater than that of TyG (*OR* = 1.58, *95%CI*: 1.24–2.03, *P* < 0.001), TyG-BMI (*OR* = 1.63, *95%CI*: 1.28–2.07, *P* < 0.001), and TyG-WC (*OR* = 1.60, *95%CI*: 1.26–2.04, *P* < 0.001). Following adjustment for gender, age, smoking, alcohol consumption, comorbidities, hypertension risk level, and physical exercise (Model 3), the Q4 group showed significantly elevated risks related to TyG, TyG-BMI, TyG-WHtR, and TyG-WC compared with the Q1 group (*OR*s for Q4 were 1.51, 1.97, 1.52, and 1.72, respectively; all *P* values< 0.01). The TyG-BMI Q4 group exhibited the most significant increase in risk in the fully adjusted Model 4 (*OR* = 2.51, *95% CI*: 1.81–3.49). However, the TyG-WHtR Q4 group exhibited a narrower confidence interval compared to other indices (TyG, TyG-BMI, TyG-WC). Overall, a progressively increasing trend in the risk of decreased eGFR was observed with higher levels of TyG-WHtR, TyG-WC, TyG-BMI, and TyG. The risk in the Q4 group for all four indices was significantly elevated compared with the Q1 group (**Table 3**).

**Table 3:** Multivariate logistic regression analysis of TyG-WHtR and its related indicators grouped by quartile in relation to decreased eGFR.

Variable	Model 1		Model 2		Model 3		Model 4	
	*OR (95%CI)*	*P* value	*OR (95%CI)*	*P* value	*OR (95%CI)*	*P* value	*OR (95%CI)*	*P* value
TyG Q1	reference	–	reference	–	reference	–	reference	–
TyG Q2	1.21 (0.94-1.57)	0.136	1.44 (1.07-1.94)	0.015	1.40 (1.04-1.89)	0.028	1.52 (1.11-2.09)	0.010
TyG Q3	1.22 (0.95-1.58)	0.119	1.21 (0.90-1.63)	0.207	1.11 (0.82-1.51)	0.484	1.43 (1.03-1.97)	0.032
TyG Q4	1.58 (1.24-2.03)	<0.001	1.77 (1.33-2.38)	<0.001	1.51 (1.11-2.04)	0.008	1.90 (1.36-2.66)	<0.001
TyG-BMI Q1	reference	–	reference	–	reference	–	reference	–
TyG-BMI Q2	0.98 (0.76-1.27)	0.895	1.16 (0.86-1.56)	0.340	1.12 (0.83-1.51)	0.470	1.28 (0.93-1.75)	0.129
TyG-BMI Q3	1.03 (0.79-1.33)	0.845	1.22 (0.91-1.64)	0.184	1.19 (0.88-1.61)	0.268	1.43 (1.04-1.97)	0.027
TyG-BMI Q4	1.63 (1.28-2.07)	<0.001	2.06 (1.55-2.73)	<0.001	1.97 (1.46-2.66)	<0.001	2.51 (1.81-3.49)	<0.001
TyG-WHtR Q1	reference	–	reference	–	reference	–	reference	–
TyG-WHtR Q2	0.97 (0.74-1.28)	0.856	0.95 (0.70-1.30)	0.761	0.93 (0.67-1.28)	0.645	1.04 (0.75-1.45)	0.814
TyG-WHtR Q3	1.44 (1.11-1.86)	0.006	1.26 (0.94-1.70)	0.129	1.21 (0.89-1.65)	0.222	1.39 (1.01-1.92)	0.047
TyG-WHtR Q4	2.18 (1.71-2.80)	<0.001	1.64 (1.23-2.20)	<0.001	1.52 (1.12-2.08)	0.008	1.84 (1.32-2.59)	<0.001
TyG-WC Q1	reference	–	reference	–	reference	–	reference	–
TyG-WC Q2	0.89 (0.68-1.16)	0.380	0.99 (0.73-1.34)	0.944	0.97 (0.71-1.32)	0.831	1.11 (0.80-1.45)	0.528
TyG-WC Q3	1.27 (0.99-1.63)	0.059	1.38 (1.03-1.84)	0.029	1.30 (0.97-1.76)	0.079	1.50 (1.10-2.06)	0.012
TyG-WC Q4	1.60 (1.26-2.04)	<0.001	1.85 (1.40-2.46)	<0.001	1.72 (1.28-2.32)	<0.001	2.28 (1.65-3.16)	<0.001

TyG, triglyceride-glucose index; TyG-BMI, triglyceride-glucose-body mass index; TyG-WC, triglyceride-glucose-waist circumference; TyG-WHtR, triglyceride-glucose-waist-to-height ratio.

This study found that although the multiplicative interaction between TyG and WHtR (*P* = 0.065) and the additive interaction (RERI = 0.334, *95%CI*: -0.170–0.837) did not reach statistical significance, the concurrent elevation of both indices (high TyG + high WHtR) was significantly associated with an increased risk of renal function decline (*OR* = 1.666, *95%CI*: 1.197–2.319), whereas an increase in either index alone did not significantly elevate the risk. This indicates that the coexistence of visceral obesity and IR constitutes a high-risk phenotype for renal function impairment, with their combined effect exceeding that of either factor alone (**Supplementary Table 9**).

### 3.3 Correlation between TyG-WHtR and its related indicators with clinical metabolic indicators

**Figure 1:**
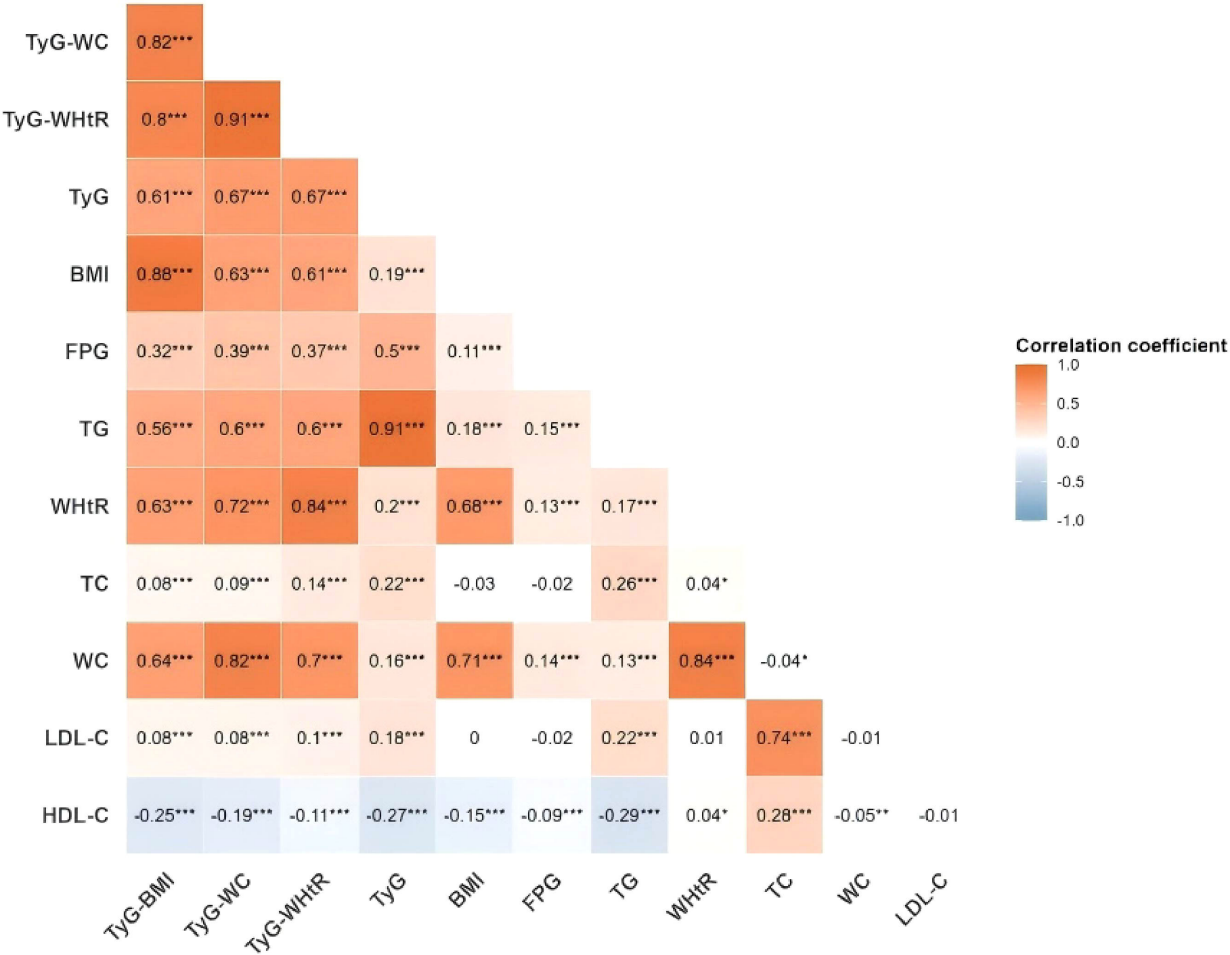
Heatmap of the correlations between TyG-WHtR and its related indices with clinical metabolic indices. WC, waist circumference; BMI, body mass index; WHtR, waist-to-height ratio; FPG, fasting plasma glucose; TC, total cholesterol; TG, triglyceride; LDL-C, low density lipoprotein; HDL-C, high density lipoprotein cholesterol; TyG, triglyceride-glucose index; TyG-BMI, triglyceride-glucose-body mass index; TyG-WC, triglyceride-glucose-waist circumference; TyG-WHtR, triglyceride-glucose-waist-to-height ratio. ^*^P < 0.05, ^**^P < 0.01, ^***^P < 0.001.

Correlation analysis revealed that TyG-WHtR and its related indices exhibited varying degrees of association with clinical metabolic indicators, with correlation coefficients ranging from very weak to very strong (r = 0.04–0.91). Correlation strength was classified as: very weak (r< 0.1), weak (r = 0.1–0.39), moderate (r = 0.40–0.69), strong (r = 0.70–0.89), and very strong (r ≥0.90). Accordingly, the TyG index demonstrated a very strong positive correlation with TG (r = 0.91, *P* < 0.001) and a weak negative correlation with HDL-C (r = −0.29, *P* < 0.001). The combined indices—TyG-WHtR, TyG-WC, and TyG-BMI—showed weak-to-strong positive correlations with TG, FPG, and anthropometric parameters (WC, WHtR, BMI) (r = 0.32–0.88, *P* < 0.001) and weak negative correlations with HDL-C (r = 0.11–0.25, *P* < 0.001). The correlation between TyG and WHtR was higher than that between TyG and BMI or WC (**Supplementary Figure 1**). Notably, TyG-WHtR correlated strongly with WHtR (r = 0.84), TyG-WC with WC (r = 0.82), and TyG-BMI with BMI (r = 0.88). The correlations between combined indices and their corresponding anthropometric measures were substantially stronger than those observed for TyG alone (e.g., TyG vs. WHtR: r = 0.20; TyG-WHtR vs. WHtR: r = 0.84). All indices, including TyG, TyG-WHtR, TyG-WC, and TyG-BMI, showed weak to very weak correlations with LDL-C and TC (r = 0.08–0.22) (**Figure 1**).

### 3.4 Associations of TyG-WHtR and related indices with eGFR decline: a logistic regression analysis with restricted cubic splines

**Figure 2:**
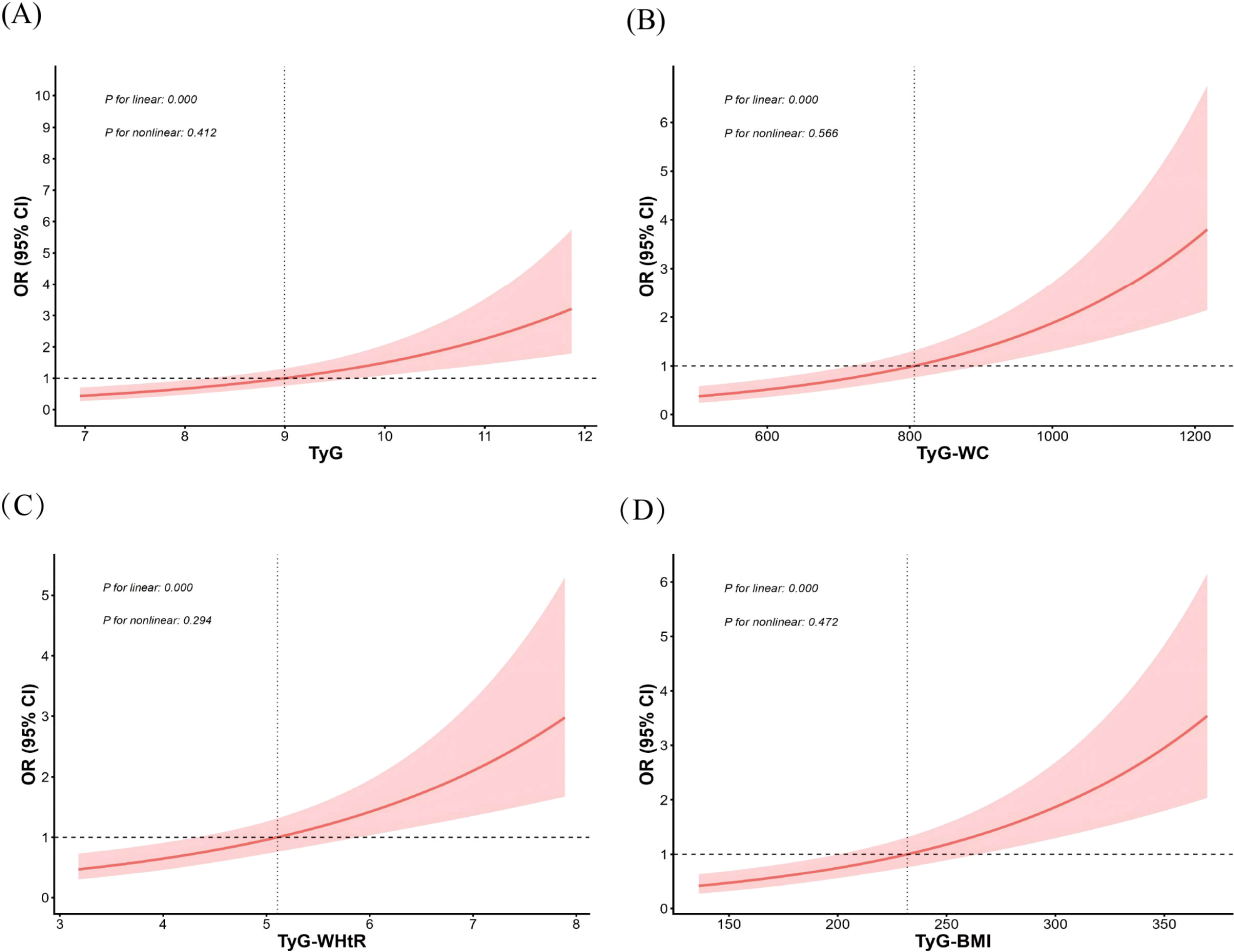
Logistic regression analysis of TyG-WHtR and its related indices with risk of eGFR decline using linear and restricted cubic spline fitting. TyG, triglyceride-glucose index; TyG-BMI, triglyceride-glucose-body mass index; TyG-WC, triglyceride-glucose-waist circumference; TyG-WHtR, triglyceride-glucose-waist-to-height ratio.

The relationship of TyG-WHtR and related indices to the risk of eGFR decline was assessed using logistic regression alongside restricted cubic spline (RCS) analysis. The results indicated that the nonlinear terms for TyG-WHtR and related indices were not statistically significant (TyG-WHtR: *P* for nonlinear = 0.294; TyG: *P* for nonlinear = 0.412; TyG-BMI: *P* for nonlinear = 0.472; TyG-WC: *P* for nonlinear = 0.566), suggesting a linear relationship. Thus, higher TyG-WHtR and related indices progressively elevated eGFR decline risk (**Figure 2**).

### 3.5 Subgroup analysis of TyG-WHtR and its associated indices with respect to the risk of eGFR decline

In subgroup analyses, a higher TyG-WHtR significantly correlated with a greater risk of eGFR decline in elderly patients with hypertension. Within each subgroup, covariates were adjusted to assess the associations of TyG-WHtR and its related indices (TyG, TyG-BMI, TyG-WC) with the risk of eGFR decline. TyG-WHtR exhibited a significant positive association across all subgroups, except in participants aged ≥80 years (*P* = 0.067) and in smokers (*P* = 0.071), where the associations were not statistically significant. The odds ratios (*OR*) ranged from 1.29 to 2.14. Moreover, significant interaction effects were observed across the age (interaction *P* < 0.001), gender(interaction *P* = 0.043), and alcohol consumption (interaction *P* < 0.001) subgroups. Specifically, a higher risk of eGFR decline associated with TyG-WHtR was found among the relatively young (65–80 years), male, and alcohol consumption in individual groups. Despite a consistent *OR* of 1.00 for TyG-WC (all *P* < 0.05), the analysis revealed statistically significant interactions, which were noted in subgroups defined by age (interaction *P* < 0.001) and alcohol consumption (interaction *P* < 0.001). Similarly, significant interactions for TyG and TyG-BMI were observed within the age (interaction *P* < 0.001) and alcohol consumption (interaction *P* < 0.001) subgroups (**Supplementary Tables 5**-**8**).

### 3.6 Predictive value of TyG-WHtR and its related indices for eGFR decline in the community elderly hypertensive population

**Figure 3:**
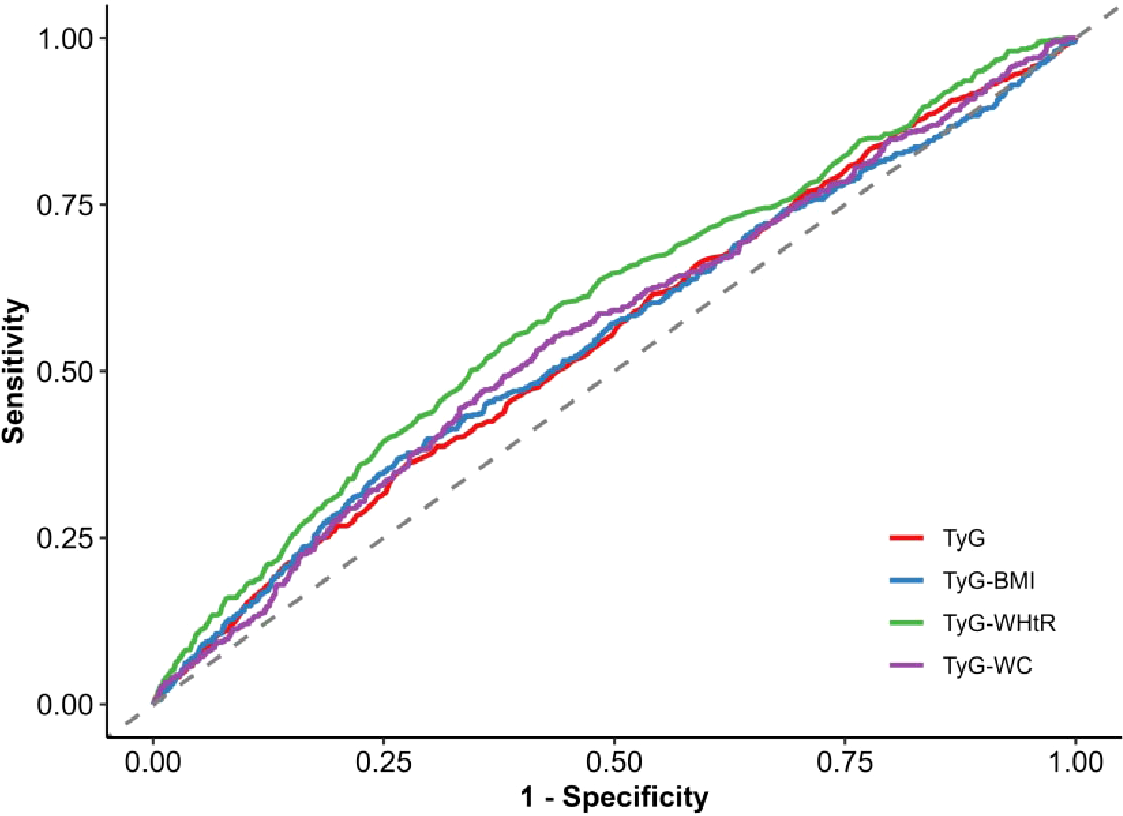
ROC curve illustrating the predictive value of TyG-WHtR and its related indices for eGFR decline in the elderly hypertensive population. TyG, triglyceride-glucose index; TyG-BMI, triglyceride-glucose-body mass index; TyG-WC, triglyceride-glucose-waist circumference; TyG-WHtR, triglyceride-glucose-waist-to-height ratio.

**Figure 4:**
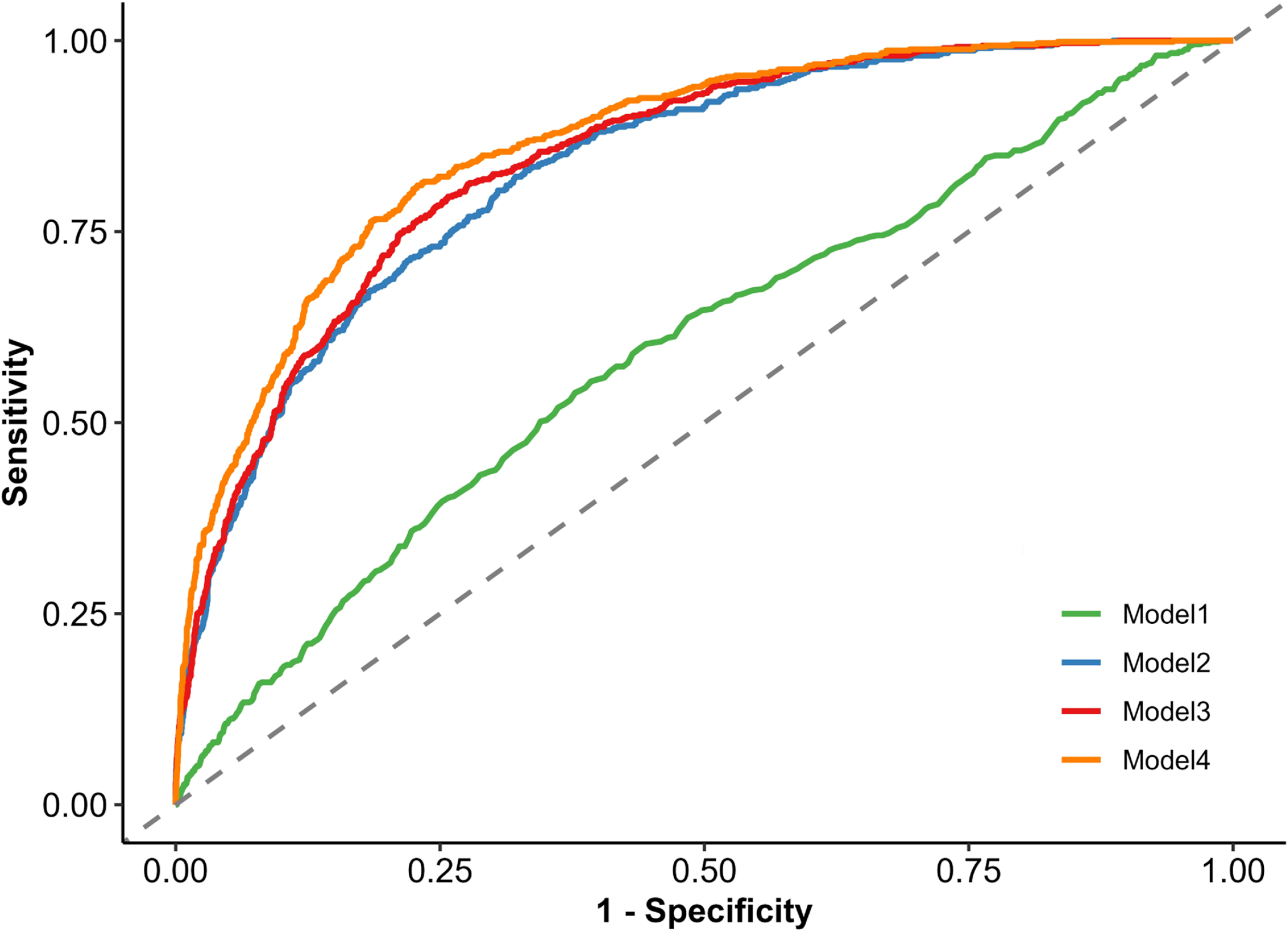
The predictive value of TyG-WHtR and its adjusted models for eGFR decline in the elderly hypertensive population as shown by the ROC curve. Model 1, unadjusted variables; Model 2, adjusted for gender and age; Model 3, adjusted for gender, age, smoking, alcohol consumption, comorbidities, hypertension risk level and physical exercise; Model 4, adjusted for gender, age, smoking, alcohol consumption, comorbidities, hypertension risk level, physical exercise, DBP, SBP, urine protein, HDL-C, TBil, ALT, AST, Hb and WBC; TyG-WHtR, triglyceride-glucose-waist-to-height ratio; eGFR, estimated glomerular filtration rate; DBP, diastolic blood pressure; SBP, systolic blood pressure; HDL-C, high density lipoprotein cholesterol; TBil, total bilirubin; ALT, alanine aminotransferase,; AST, aspartate aminotransferase; Hb, hemoglobin; WBC, white blood cell.

The occurrence of eGFR decline in the community elderly hypertensive population aged ≥65 years was used as the dependent variable (“0” = eGFR normal, “1” = eGFR decline), while the TyG-WHtR and its related indices levels in this population served as the test variables. The ROC curve was then plotted (**Figure 3**). Of the four indices assessed—TyG-WHtR, TyG, TyG-BMI, and TyG-WC, TyG-WHtR emerged as the top performer. It achieved the highest area under the curve (AUC = 0.596; *95% CI*: 0.572–0.621), and its ROC curve was positioned closest to the upper-left corner, corresponding to a sensitivity of 0.539 and a specificity of 0.622. This indicates the most favorable overall balance between sensitivity and specificity, and predictive efficacy significantly exceeded that of TyG, TyG-BMI, and TyG-WC. In comparison, TyG-BMI (AUC = 0.549), TyG-WC (AUC = 0.557), and TyG (AUC = 0.550) exhibited similar performance, with no significant differences among them (all *P* > 0.05). These results suggest that TyG-WHtR provides superior predictive ability for eGFR decline in the elderly hypertensive community population, whereas TyG-BMI and TyG-WC offer no significant improvement over TyG (**Figure 3** and **Table 4**). The predictive performance of four stepwise-adjusted models based on TyG-WHtR was also compared. Model 4 exhibited the best predictive performance, with its ROC curve positioned nearest to the top-left corner. This reflects an optimal trade-off between sensitivity (0.810) and specificity (0.771). The model also showed the highest discriminative ability, achieving an AUC of 0.864 (*95% CI*: 0.849–0.879). ROC curves from Model 1 to Model 4 showed a progressive shift toward the upper-left corner, reflecting continuous improvement in predictive performance. Both as an individual index and within Model 4, TyG-WHtR consistently exhibited the highest predictive value, confirming its significant role in predicting eGFR decline among the community elderly hypertensive population aged ≥65 years (**Figure 4** and **Tables 4**, **5**).

**Table 4:** The predictive value of TyG-WHtR and its related indices for renal function decline in the elderly hypertensive population.

Variable	Optimal cut-off value	Sensitivity	Specificity	AUC (*95%CI*)	*P*_1_	*P*_2_
TyG	9.245	0.361	0.724	0.55 (0.525,0.575)	<0.001	<NA>
TyG-BMI	250.88	0.369	0.734	0.549 (0.523,0.575)	<0.001	0.886
TyG-WHtR	5.115	0.539	0.622	0.596 (0.572,0.621)	<0.001	<0.001
TyG-WC	810.22	0.544	0.573	0.557 (0.532,0.583)	<0.001	0.509

TyG, triglyceride-glucose index; TyG-BMI, triglyceride-glucose-body mass index; TyG-WC, triglyceride-glucose-waist circumference; TyG-WHtR, triglyceride-glucose-waist-to-height ratio; *P*_1_ represents the test result of the area under the curve compared to 0.5; *P*_2_ shows the comparison of the TyG with other insulin resistance indices based on the area under the curve.

**Table 5:** The predictive value of TyG-WHtR adjusted Model for renal function decline in the elderly hypertensive population.

Model	Optimal cut-off value	Sensitivity	Specificity	AUC (*95%CI*)	*P*_1_	*P*_2_
Model1	0.165	0.539	0.622	0.596 (0.572,0.621)	<0.001	<NA>
Model2	0.118	0.822	0.679	0.832 (0.816,0.849)	<0.001	<0.001
Model3	0.144	0.796	0.742	0.843 (0.828,0.859)	<0.001	<0.001
Model4	0.151	0.810	0.771	0.864 (0.849,0.879)	<0.001	<0.001

Model 1, unadjusted variables; Model 2, adjusted for gender and age; Model 3, adjusted for gender, age, smoking, alcohol consumption, comorbidities, hypertension risk level and physical exercise; Model 4, adjusted for gender, age, smoking, alcohol consumption, comorbidities, hypertension risk level, physical exercise, DBP, SBP, urine protein, HDL-C, TBil, ALT, AST, Hb and WBC; TyG-WHtR, triglyceride-glucose-waist-to-height ratio; eGFR, estimated glomerular filtration rate; DBP, diastolic blood pressure; SBP, systolic blood pressure; HDL-C, high density lipoprotein cholesterol; TBil, total bilirubin; ALT, alanine aminotransferase,; AST, aspartate aminotransferase; Hb, hemoglobin; WBC, white blood cell. *P*_1_ indicates the test result of the area under the curve against 0.5; *P*_2_ shows the comparison of the area under the curve for Model 1 with those of other models.

## 4 Discussion

As the population ages, the prevalence of hypertension among older adults has continued to rise (16). United Nations data from 2019 indicates that the world’s population of 65 years and older stood at 703 million, accounting for 9% of the total, and is anticipated to rise to 16% by 2050 (17). In China, hypertension affects 44.7% of adults aged 35–75, with individuals aged 55 and above constituting 72.9% of the cases. Moreover, the disease burden of severe hypertension is primarily concentrated in individuals aged ≥65 years (18). Despite the demonstrated efficacy of antihypertensive therapy and lifestyle interventions, global hypertension control rates remain low, particularly in low-income countries, where awareness, treatment, and control rates are markedly inferior to those in high-income regions (2). Long-term poor blood pressure control can lead to damage in vital organs, including the heart, brain, kidneys, and peripheral vasculature. In recent years, numerous novel IR indices have been identified to be associated with renal impairment, such as TyG and TyG-WC (13). This study investigated the association between the TyG-WHtR index—a novel marker of insulin resistance combined with central obesity—and renal function decline in elderly individuals with hypertension.

The decline in eGFR correlated with significantly elevated levels of the TyG-WHtR index and its related metrics (TyG, TyG-WC, TyG-BMI) compared to the normal eGFR cohort. TyG-WHtR was found to be closely correlated with obesity parameters and TG. The analysis identified TyG-WHtR and its associated indices as independent predictors of declining eGFR. Furthermore, a graded, dose-response relationship was observed, wherein higher values corresponded to a progressively greater risk of renal function deterioration. In Model 1, TyG-WHtR demonstrated the strongest association and predictive robustness. After adjustment for confounding factors, this association remained statistically significant. Further findings from the quartile analysis revealed that the risk in the Q3 group of TyG-WHtR within Model 1 increased markedly, suggesting an earlier warning potential. Although TyG-BMI exhibited a higher OR value in the Q4 group of Model 4, its confidence interval was broader than that of TyG-WHtR, indicating lower stability compared with TyG-WHtR. This study found through four-category analysis that the risk of kidney function impairment significantly increased when both TyG and WHtR were elevated simultaneously, whereas an increase in either indicator alone did not show a significant risk. Although the interaction did not reach statistical significance, the risk was significantly increased when both factors coexisted, indicating a stronger warning value in clinical assessment compared to abnormalities in single indices. The predictive performance of TyG-WHtR for the risk of eGFR decline among the community elderly hypertensive population was found to be the strongest compared with the other three indices. In Model 4, the predictive efficacy of TyG-WHtR for eGFR decline reached its highest level (AUC = 0.864).

The significant predictive value of TyG-WHtR for renal function decline in the community elderly hypertensive population was demonstrated in this study. However, the precise biological mechanism linking TyG-WHtR to eGFR decline remains unclear. This may be attributable to the synergistic effects of central obesity and IR on renal function. It is well-established that obesity is closely linked to IR. Recent Mendelian randomization studies have provided further evidence, confirming that obesity—as indicated by BMI and WC—directly contributes to the decline of renal function and increases the risk of developing various kidney diseases, including CKD, hypertensive nephropathy, and diabetic nephropathy. While the impact of obesity on kidney function is partly attribu5to high blood pressure and type 2 diabetes, additional pathways involving immune dysregulation, metabolic disturbance, and extracellular matrix remodeling also contribute (19). Under obese conditions, infiltration and M1-type polarization of macrophages within visceral adipose tissue represent the core heart mechanism driving chronic inflammation. M1 macrophage-derived pro-inflammatory cytokines, primarily IL-1β and TNF-α, activate the NF-κB pathway in intrinsic renal cells. Following this activation, the upregulation of chemokines (such as MCP-1 and IL-8) and adhesion molecules (including ICAM-1 and VCAM-1) occurs. The resulting molecules mediate the infiltration of inflammatory cells—primarily neutrophils and monocytes/macrophages—into the kidney, a process that exacerbates renal injury (20). IR has been shown to induce down regulation of the PPAR/PPARGC1A signaling pathway in renal tubular cells, resulting in a significant reduction in fatty acid β-oxidation capacity and subsequently promoting lipid accumulation, apoptosis, and fibrosis. Meanwhile, enhanced glycolysis is observed in podocytes under IR conditions (e.g., upregulation of PFKFB3), which further promotes inflammatory and fibrotic processes (21, 22). In obese individuals, the level of adipose tissue–derived complement C1q/tumor necrosis factor–related protein 3 (CTRP3) is markedly reduced (23). The down regulation of CTRP3 suppresses the AMPK signaling pathway, simultaneously triggering two interrelated pathological processes—disordered lipid metabolism and necrotic inflammation—that collectively drive the progression of renal tubular injury (24). Moreover, obesity can induce glomerular hyperfiltration and compensatory hypertrophy, resulting in a relative dilution and decreased density of non-proliferative podocytes. This disruption of the filtration barrier leads to proteinuria and glomerulosclerosis, initiating a vicious cycle of progressive decline in renal function (25).

In addition to the indirect effects mediated by obesity mentioned above, IR and its compensatory hyperinsulinemia can also exacerbate hypertension and renal impairment through multiple mechanisms. First, regarding blood pressure regulation, hyperinsulinemia can directly elevate blood pressure by activating the sympathetic nervous system and promoting sodium reabsorption in the renal tubules (26). Hyperinsulinemia primarily enhances sodium reabsorption by stimulating the sodium-hydrogen exchanger 3 (NHE3) located on the apical membrane of the proximal tubules in the kidney, thereby leading to sodium and water retention as well as elevated blood pressure (27). Simultaneously, hyperinsulinemia induces vasoconstriction and endothelial dysfunction by stimulating the release of endothelin-1 (ET-1) and inhibiting the activity of endothelial nitric oxide synthase (eNOS), thereby increasing peripheral vascular resistance (28, 29). In addition, insulin can enhance sympathetic nerve activity by acting on the arcuate nucleus of the hypothalamus, thereby further exacerbating vasoconstriction and increasing blood pressure (30). Secondly, in terms of kidney injury, the imbalance of residual insulin signal transduction under IR conditions can directly trigger pathological changes in kidney cells (21). Under IR conditions, hyperinsulinemia directly acts on glomerular podocytes by promoting insulin receptor degradation and inhibiting downstream Akt and MAPK signaling pathways. This leads to reduced glucose uptake and impaired actin cytoskeletal remodeling in podocytes. The resulting signaling impairment further disrupts cytoskeletal integrity, causing foot process effacement, apoptosis, and a decrease in podocyte number. Consequently, the function of the glomerular filtration barrier is compromised, ultimately leading to the development of proteinuria and progressive renal function impairment (31, 32). Under high glucose conditions, glucose uptake mediated by GLUT1 can upregulate renin receptor expression and reactive oxygen species generation, thereby inducing the expression of TGF-β1 and CTGF in renal medullary collecting duct cells, which promotes the progression of renal tubular fibrosis (33). Furthermore, IR increases peripheral lipolysis of free fatty acids, upregulates renal tubular CD36 uptake, and inhibits PPAR-α/PGC-1α-mediated fatty acid oxidation, resulting in ectopic lipid deposition in the kidney. This process, driven by metabolic dysfunction, can, to some extent, occur independently of systemic obesity and directly induce endoplasmic reticulum stress and apoptosis (34). In summary, IR and hyperinsulinemia accelerate the decline of renal function through mechanisms such as promoting sodium retention, vasoconstriction, fibrosis, inducing podocyte injury, and lipotoxicity, which together with the indirect effects of obesity contribute to this process.

Significant interactions were found among three subgroups in the detailed analysis: age, gender, and alcohol consumption. The TyG-WHtR relationship with declining eGFR varied significantly across these subgroups. The risk associated with TyG-WHtR was higher in males than in females, a finding consistent with prior studies indicating sex-based differences in its association with kidney disease risk (35). Mechanistically, the faster progression of renal injury in males may be attributed to androgen-mediated metabolic reprogramming, which results in an overactive tricarboxylic acid (TCA) cycle and increased oxidative stress. In contrast, females tend to accumulate more pyruvate with antioxidant properties in the kidney, helping to maintain redox homeostasis to some extent (36). On the other hand, this difference may stem from sex-specific patterns of fat distribution; males tend to exhibit abdominal obesity and visceral fat accumulation, which are more strongly linked to IR and metabolic syndrome, whereas in females, fat is mainly distributed subcutaneously, providing a certain degree of metabolic protection before menopause (37). Therefore, in clinical practice, greater attention should be given to the management of abdominal obesity and IR in male hypertension patients. Additionally, age serves as a significant effect modifier. A stronger risk association between TyG-WHtR and eGFR decline was observed among individuals with lower age (65–80 years), whereas in those with higher age (age ≥ 80 years), this association did not reach statistical significance. The underlying reasons for this difference remain uncertain. The decline in renal function among older individuals is likely driven primarily by intrinsic physiological processes associated with age itself, such as the irreversible loss of nephrons and renal vascular sclerosis (38). These non-metabolic factors may partly obscure or weaken the impact of metabolic risk factors—represented by IR and central obesity and reflected by TyG-WHtR—on renal function. As a result, no significant association was detected between TyG-WHtR and the risk of eGFR decline among individuals aged ≥80 years. Third, in alcohol consumption individuals, an elevated TyG-WHtR was linked to a substantially higher risk of eGFR decline (*OR* = 2.14) than in non-alcohol-consuming individuals (*OR* = 1.45). This may be explained by the fact that alcohol intake can aggravate IR, enhance hepatic lipid synthesis, and increase oxidative stress, thereby producing a synergistic effect with the high metabolic load indicated by TyG-WHtR, ultimately accelerating renal injury (39).

Currently, the IR indicators used in clinical practice include not only the traditional fasting insulin, HOMA-IR index, and oral glucose tolerance test (OGTT), but also several novel IR indicators derived from anthropometric and laboratory parameters, such as TyG, TyG-BMI, TyG-WHtR, and TyG-WC. The predictive value of TyG and its related indices has been demonstrated in a variety of diseases. In an extensive prospective cohort study conducted among the U.S. population with metabolic syndrome, TyG-WHtR exhibited significantly greater predictive power for all-cause mortality, cardiovascular mortality, and diabetes mortality, with its performance for cardiovascular mortality surpassing that of the single TyG index (40). Another investigation based on the U.S. NHANES database further demonstrated that TyG-WHtR served as a superior indicator compared with the TyG index in predicting all-cause mortality, cardiovascular disease mortality, and chronic heart failure among patients with non-alcoholic fatty liver disease (NAFLD) (41). Among middle-aged and elderly individuals, higher TyG-WHtR levels were found to be independently associated with an increased risk of cardiovascular disease events (42). In patients with hypertension, the TyG index and its derivatives (TyG-BMI, TyG-WC, and TyG-WHtR) were identified as independent predictors of all-cause mortality and cardiovascular mortality, with TyG-WHtR showing the strongest correlation. For each 1-unit increase in TyG-WHtR, the risk of all-cause mortality and cardiovascular mortality rose by 41.7% and 48.1%, respectively (43). The present results align with earlier investigations, demonstrating that TyG-WHtR offers stronger clinical predictive ability than TyG, TyG-BMI, and TyG-WC.

This study has a number of limitations. First, its cross-sectional design relied on standardized questionnaires for medical history and comorbidities, introducing potential recall bias. Second, due to the cross-sectional design and the absence of longitudinal follow-up data, this study could not investigate causal relationships between TyG-WHtR and eGFR decline. Therefore, future longitudinal cohort studies are warranted to explore temporal relationships and potential causal mechanisms. Third, despite adjusting for numerous confounders, residual confounding cannot be excluded. Fourth, the study population was limited to Huaian City, Jiangsu Province, and consisted exclusively of elderly Chinese participants; this limits the generalizability of our findings to younger populations or other ethnic groups. Therefore, future multicenter studies with larger, more diverse cohorts are warranted to corroborate the results. Fifth, the lack of data on the utilization of hypoglycemic and lipid-lowering agents. Such medications may reduce the TyG index, potentially causing its effect on renal function to be underestimated. Nevertheless, this point also indirectly implies that TyG could retain predictive value even under pharmacological treatment.

## 5 Conclusions

In summary, elevated levels of the TyG-WHtR index and its derivatives independently predicted a higher risk of eGFR decline in community-based hypertensive patients aged ≥65 years, demonstrating a positive dose-response relationship. Among these indicators, TyG-WHtR exhibited a stronger predictive capacity for eGFR decline than TyG, TyG-WC, and TyG-BMI. In individuals aged 65–80 years, male, and those with alcohol consumption habits, a significant increase in the risk of eGFR decline was observed with higher TyG-WHtR levels. Given its accessibility in primary healthcare settings, TyG-WHtR may serve as a valuable early warning tool for detecting renal function impairment in elderly hypertensive populations.

## Data Availability

The raw data supporting the conclusions of this article will be made available by the corresponding author upon reasonable request.
