# Supplementary material for: The triglyceride-glucose–waist-to-height ratio is the strongest predictor of reduced kidney function in elderly hypertensive patients
Source: Front Endocrinol (Lausanne). 2026 Jun 15;17:1789763. doi: 10.3389/fendo.2026.1789763 (PMC13310771; doi:10.3389/fendo.2026.1789763)
Supplement: Supplementary file 1 [file DataSheet1.docx]

Supplementary Material

**Supplementary Table 1**

Independent variable multicollinearity test for TyG Model 3 and Model 4

|  | Model 3 VIF | Model 4 VIF |
| --- | --- | --- |
| TyG | 1.087 | 1.254 |
| Gender | 1.350 | 1.710 |
| Age | 1.028 | 1.159 |
| Smoking | 1.209 | 1.237 |
| Alcohol consumption | 1.249 | 1.270 |
| Comorbidity | 3.187 | 3.661 |
| Hypertension risk level | 3.218 | 3.867 |
| Physical exercise | 1.018 | 1.030 |
| SBP | / | 1.633 |
| DBP | / | 1.496 |
| Hb | / | 1.490 |
| WBC | / | 1.105 |
| AST | / | 2.228 |
| ALT | / | 2.369 |
| Urine protein | / | 1.063 |
| HDL-C | / | 1.117 |
| TBil | / | 1.111 |

TyG, triglyceride-glucose index; SBP, systolic blood pressure; DBP, diastolic blood pressure; Hb, hemoglobin; WBC, white blood cell; ALT, alanine aminotransferase; AST, aspartate aminotransferase; TBil, total bilirubin; HDL-C, high density lipoprotein cholesterol; VIF: variance inflation factor.

**Supplementary Table 2**

Independent variable multicollinearity test for TyG-WC Model 3 and Model 4

|  | Model 3 VIF | Model 4 VIF |
| --- | --- | --- |
| TyG-WC | 1.139 | 1.263 |
| Gender | 1.322 | 1.635 |
| Age | 1.027 | 1.162 |
| Smoking | 1.209 | 1.239 |
| Alcohol consumption | 1.254 | 1.276 |
| Comorbidity | 3.236 | 3.742 |
| Hypertension risk level | 3.482 | 4.202 |
| Physical exercise | 1.018 | 1.031 |
| SBP | / | 1.651 |
| DBP | / | 1.497 |
| Hb | / | 1.479 |
| WBC | / | 1.092 |
| AST | / | 2.231 |
| ALT | / | 2.380 |
| Urine protein | / | 1.060 |
| HDL-C | / | 1.092 |
| TBil | / | 1.107 |

TyG-WC, triglyceride-glucose-waist circumference; SBP, systolic blood pressure; DBP, diastolic blood pressure; Hb, hemoglobin; WBC, white blood cell; ALT, alanine aminotransferase; AST, aspartate aminotransferase; TBil, total bilirubin; HDL-C, high density lipoprotein cholesterol. VIF: variance inflation factor.

**Supplementary Table 3**

Independent variable multicollinearity test for TyG-WHtR Model 3 and Model 4

|  | Model 3 VIF | Model 4 VIF |
| --- | --- | --- |
| TyG-WHtR | 1.243 | 1.353 |
| Gender | 1.419 | 1.789 |
| Age | 1.031 | 1.173 |
| Smoking | 1.209 | 1.239 |
| Alcohol consumption | 1.252 | 1.274 |
| Comorbidity | 3.253 | 3.757 |
| Hypertension risk level | 3.504 | 4.214 |
| Physical exercise | 1.019 | 1.032 |
| SBP | / | 1.644 |
| DBP | / | 1.495 |
| Hb | / | 1.476 |
| WBC | / | 1.096 |
| AST | / | 2.228 |
| ALT | / | 2.366 |
| Urine protein | / | 1.059 |
| HDL-C | / | 1.088 |
| TBil | / | 1.107 |

TyG-WHtR, triglyceride-glucose-waist-to-height ratio; SBP, systolic blood pressure; DBP, diastolic blood pressure; Hb, hemoglobin; WBC, white blood cell; ALT, alanine aminotransferase; AST, aspartate aminotransferase; TBil, total bilirubin; HDL-C, high density lipoprotein cholesterol. VIF: variance inflation factor.

**Supplementary Table 4**

Independent variable multicollinearity test for TyG-BMI Model 3 and Model 4

|  | Model 3 VIF | Model 4 VIF |
| --- | --- | --- |
| TyG-BMI | 1.166 | 1.319 |
| Gender | 1.329 | 1.668 |
| Age | 1.033 | 1.160 |
| Smoking | 1.211 | 1.243 |
| Alcohol consumption | 1.250 | 1.271 |
| Comorbidity | 3.301 | 3.839 |
| Hypertension risk level | 3.591 | 4.349 |
| Physical exercise | 1.018 | 1.030 |
| SBP | / | 1.646 |
| DBP | / | 1.494 |
| Hb | / | 1.488 |
| WBC | / | 1.092 |
| AST | / | 2.227 |
| ALT | / | 2.365 |
| Urine protein | / | 1.058 |
| HDL-C | / | 1.117 |
| TBil | / | 1.107 |

TyG-BMI, triglyceride-glucose-body-mass index; SBP, systolic blood pressure; DBP, diastolic blood pressure; Hb, hemoglobin; WBC, white blood cell; ALT, alanine aminotransferase; AST, aspartate aminotransferase; TBil, total bilirubin; HDL-C, high density lipoprotein cholesterol. VIF: variance inflation factor.

**Supplementary Table 5**

Subgroup analysis and interaction test of TyG-WHtR

| Subground | Number | *OR* | *95%CI* | *P* value | *P* for interaction |
| --- | --- | --- | --- | --- | --- |
| Gender |  |  |  |  |  |
| Female | 1872 | 1.49 | (1.21, 1.82) | <0.001 | 0.043 |
| Male | 1893 | 1.55 | (1.15, 2.07) | 0.004 |  |
| Age |  |  |  |  |  |
| 65~80 years | 3172 | 1.61 | (1.31, 1.97) | <0.001 | <0.001 |
| ≥80 years | 593 | 1.30 | (0.98, 1.73) | 0.067 |  |
| Hypertension risk level |  |  |  |  |  |
| Moderate risk | 1595 | 1.59 | (1.16, 2.18) | 0.004 | 0.470 |
| High risk | 761 | 1.85 | (1.26, 2.74) | 0.002 |  |
| Very high risk | 1409 | 1.29 | (1.03, 1.63) | 0.030 |  |
| Smoking |  |  |  |  |  |
| No | 3295 | 1.48 | (1.25, 1.76) | <0.001 | 0.300 |
| Yes | 470 | 2.04 | (0.94, 4.46) | 0.071 |  |
| Alcohol consumption |  |  |  |  |  |
| No | 2832 | 1.45 | (1.22, 1.74) | <0.001 | <0.001 |
| Yes | 933 | 2.14 | (1.47, 3.10) | <0.001 |  |
| Comorbidity |  |  |  |  |  |
| No | 2525 | 1.61 | (1.28, 2.03) | <0.001 | 0.187 |
| Yes | 1240 | 1.35 | (1.06, 1.72) | 0.014 |  |
| Urine protein |  |  |  |  |  |
| No | 2940 | 1.40 | (1.14, 1.72) | 0.001 | 0.434 |
| Yes | 825 | 1.64 | (1.23, 2.19) | <0.001 |  |
| Physical exercise |  |  |  |  |  |
| No | 1883 | 1.54 | (1.24, 1.93) | <0.001 | 0.735 |
| Yes | 1882 | 1.45 | (1.13, 1.86) | 0.004 |  |

TyG-WHtR, triglyceride-glucose-waist-to-height ratio.

**Supplementary Table 6**

Subgroup analysis and interaction test of TyG-WC

| Subground | Number | *OR* | *95%CI* | *P* value | *P* for interaction |
| --- | --- | --- | --- | --- | --- |
| Gender |  |  |  |  |  |
| Female | 1872 | 1.00 | (1.00, 1.00) | <0.001 | 0.290 |
| Male | 1893 | 1.00 | (1.00, 1.00) | 0.005 |  |
| Age |  |  |  |  |  |
| 65~80 years | 3172 | 1.00 | (1.00, 1.00) | <0.001 | <0.001 |
| ≥80 years | 593 | 1.00 | (1.00, 1.00) | 0.032 |  |
| Hypertension risk level |  |  |  |  |  |
| Moderate risk | 1595 | 1.00 | (1.00, 1.00) | 0.018 | 0.948 |
| High risk | 761 | 1.00 | (1.00, 1.01) | 0.003 |  |
| Very high risk | 1409 | 1.00 | (1.00, 1.00) | 0.004 |  |
| Smoking |  |  |  |  |  |
| No | 3295 | 1.00 | (1.00, 1.00) | <0.001 | 0.369 |
| Yes | 470 | 1.00 | (1.00, 1.01) | 0.038 |  |
| Alcohol consumption |  |  |  |  |  |
| No | 2832 | 1.00 | (1.00, 1.00) | <0.001 | <0.001 |
| Yes | 933 | 1.00 | (1.00, 1.01) | 0.010 |  |
| Comorbidity |  |  |  |  |  |
| No | 2525 | 1.00 | (1.00, 1.00) | <0.001 | 0.455 |
| Yes | 1240 | 1.00 | (1.00, 1.00) | 0.002 |  |
| Urine protein |  |  |  |  |  |
| No | 2940 | 1.00 | (1.00, 1.00) | 0.001 | 0.316 |
| Yes | 825 | 1.00 | (1.00, 1.01) | <0.001 |  |
| Physical exercise |  |  |  |  |  |
| No | 1883 | 1.00 | (1.00, 1.00) | <0.001 | 0.688 |
| Yes | 1882 | 1.00 | (1.00, 1.00) | 0.002 |  |

TyG-WC, triglyceride-glucose-waist circumference.

**Supplementary Table 7**

Subgroup analysis and interaction test of TyG

| Subground | Number | *OR* | *95%CI* | *P* value | *P* for interaction |
| --- | --- | --- | --- | --- | --- |
| Gender |  |  |  |  |  |
| Female | 1872 | 1.41 | (1.14, 1.74) | 0.002 | 0.346 |
| Male | 1893 | 1.24 | (0.93, 1.63) | 0.138 |  |
| Age |  |  |  |  |  |
| 65~80 years | 3172 | 1.43 | (1.16, 1.74) | <0.001 | <0.001 |
| ≥80 years | 593 | 1.17 | (0.87, 1.58) | 0.287 |  |
| Hypertension risk level |  |  |  |  |  |
| Moderate risk | 1595 | 1.34 | (0.99, 1.80) | 0.056 | 0.156 |
| High risk | 761 | 1.63 | (1.09, 2.43) | 0.016 |  |
| Very high risk | 1409 | 1.16 | (0.92, 1.47) | 0.214 |  |
| Smoking |  |  |  |  |  |
| No | 3295 | 1.29 | (1.08, 1.53) | 0.005 | 0.070 |
| Yes | 470 | 2.04 | (1.10, 3.83) | 0.024 |  |
| Alcohol consumption |  |  |  |  |  |
| No | 2832 | 1.33 | (1.11, 1.59) | 0.002 | <0.001 |
| Yes | 933 | 1.36 | (0.95, 1.92) | 0.084 |  |
| Comorbidity |  |  |  |  |  |
| No | 2525 | 1.40 | (1.11, 1.75) | 0.004 | 0.336 |
| Yes | 1240 | 1.21 | (0.95, 1.55) | 0.124 |  |
| Urine protein |  |  |  |  |  |
| No | 2940 | 1.16 | (0.94, 1.43) | 0.159 | 0.079 |
| Yes | 825 | 1.73 | (1.30, 2.31) | <0.001 |  |
| Physical exercise |  |  |  |  |  |
| No | 1883 | 1.24 | (0.99, 1.56) | 0.063 | 0.288 |
| Yes | 1882 | 1.47 | (1.15, 1.88) | 0.002 |  |

TyG, triglyceride-glucose index.

**Supplementary Table 8**

Subgroup analysis and interaction test of TyG-BMI

| Subground | Number | *OR* | *95%CI* | *P* value | *P* for interaction |
| --- | --- | --- | --- | --- | --- |
| Gender |  |  |  |  |  |
| Female | 1872 | 1.01 | (1.00, 1.01) | <0.001 | 0.650 |
| Male | 1893 | 1.00 | (1.00, 1.01) | 0.187 |  |
| Age |  |  |  |  |  |
| 65~80 years | 3172 | 1.01 | (1.00, 1.01) | <0.001 | <0.001 |
| ≥80 years | 593 | 1.00 | (1.00, 1.01) | 0.072 |  |
| Hypertension risk level |  |  |  |  |  |
| Moderate risk | 1595 | 1.00 | (1.00, 1.01) | 0.143 | 0.963 |
| High risk | 761 | 1.01 | (1.00, 1.01) | 0.054 |  |
| Very high risk | 1409 | 1.01 | (1.00, 1.01) | 0.003 |  |
| Smoking |  |  |  |  |  |
| No | 3295 | 1.01 | (1.00, 1.01) | <0.001 | 0.566 |
| Yes | 470 | 1.01 | (0.99, 1.02) | 0.293 |  |
| Alcohol consumption |  |  |  |  |  |
| No | 2832 | 1.01 | (1.00, 1.01) | <0.001 | <0.001 |
| Yes | 933 | 1.00 | (1.00, 1.01) | 0.445 |  |
| Comorbidity |  |  |  |  |  |
| No | 2525 | 1.01 | (1.00, 1.01) | 0.005 | 0.438 |
| Yes | 1240 | 1.01 | (1.00, 1.01) | 0.004 |  |
| Urine protein |  |  |  |  |  |
| No | 2940 | 1.00 | (1.00, 1.01) | 0.013 | 0.228 |
| Yes | 825 | 1.01 | (1.00, 1.01) | 0.001 |  |
| Physical exercise |  |  |  |  |  |
| No | 1883 | 1.01 | (1.00, 1.01) | <0.001 | 0572 |
| Yes | 1882 | 1.01 | (1.00, 1.01) | <0.001 |  |

TyG-BMI, triglyceride-glucose-body mass index.

**Supplementary Table 9**

Interaction analysis of TyG and WHtR on the risk of renal function decline

|  | *OR (95%CI)* | *P value* |
| --- | --- | --- |
| low TyG + low WHtR | reference | - |
| high TyG+ low WHtR | 1.097 (0.755–1.594) | 0.627 |
| high WHtR+low TyG | 1.236 (0.892–1.713) | 0.203 |
| high TyG +high WHtR | 1.666 (1.197–2.319) | 0.002 |
| Multiplicative interaction | - | 0.065 |
| Additive interaction (RERI) | 0.334 (–0.170 to 0.837) | - |

Models were adjusted for gender, age, smoking, alcohol consumption, comorbidities, hypertension risk level, physical exercise, DBP, SBP, urine protein, HDL-C, TBil, ALT, AST, Hb and WBC; DBP, diastolic blood pressure; SBP, systolic blood pressure; HDL-C, high density lipoprotein cholesterol; TBil, total bilirubin; ALT, alanine aminotransferase; AST, aspartate aminotransferase; Hb, hemoglobin; WBC, white blood cell; TyG, triglyceride-glucose index; WHtR, waist-to-height ratio; Statistical significance of multiplicative interaction was assessed by the P value. For additive interaction , statistical significance is determined by whether the 95% confidence interval excludes 0.


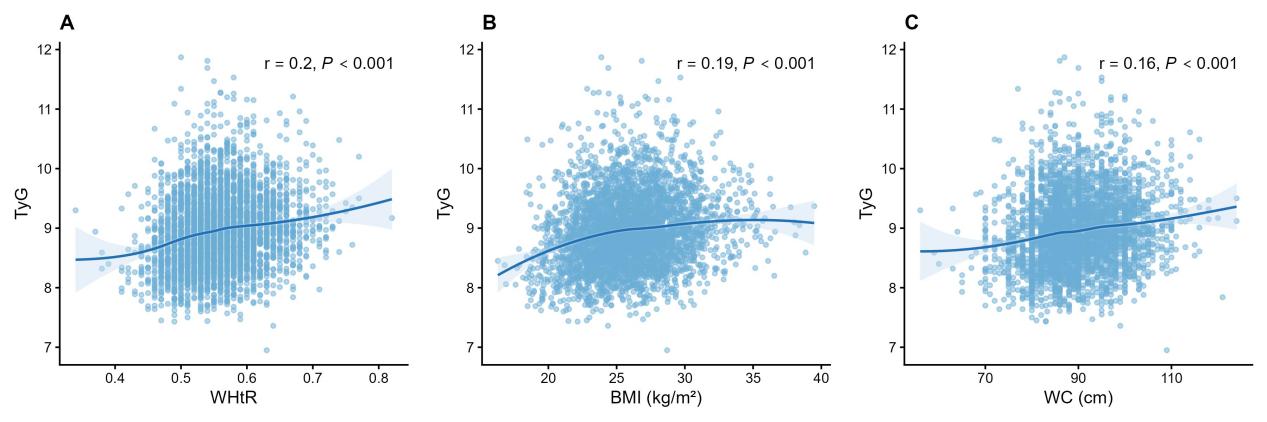
**Supplementary Fig. 1. Comparison of the correlation between TyG and WHtR, BMI, and WC**

Correlation analyses between triglyceride-glucose index (TyG) and anthropometric indices, including waist-to-height ratio (WHtR) (A), body mass index (BMI) (B), and waist circumference (WC) (C).

TyG, triglyceride-glucose index; WHtR, waist-to-height ratio; BMI, body mass index ; WC, waist circumference.
